# Variable Selection Using Nonlocal Priors in High-Dimensional Generalized Linear Models With Application to fMRI Data Analysis

**DOI:** 10.3390/e22080807

**Published:** 2020-07-23

**Authors:** Xuan Cao, Kyoungjae Lee

**Affiliations:** 1 Department of Mathematical Sciences, University of Cincinnati, Cincinnati, OH 45221, USA; caox4@ucmail.uc.edu; 2 Department of Statistics, Inha University, Incheon 22212, Korea

**Keywords:** high-dimensional, nonlocal prior, strong selection consistency

## Abstract

High-dimensional variable selection is an important research topic in modern statistics. While methods using nonlocal priors have been thoroughly studied for variable selection in linear regression, the crucial high-dimensional model selection properties for nonlocal priors in generalized linear models have not been investigated. In this paper, we consider a hierarchical generalized linear regression model with the product moment nonlocal prior over coefficients and examine its properties. Under standard regularity assumptions, we establish strong model selection consistency in a high-dimensional setting, where the number of covariates is allowed to increase at a sub-exponential rate with the sample size. The Laplace approximation is implemented for computing the posterior probabilities and the shotgun stochastic search procedure is suggested for exploring the posterior space. The proposed method is validated through simulation studies and illustrated by a real data example on functional activity analysis in fMRI study for predicting Parkinson’s disease.

## 1. Introduction

With the increasing ability to collect and store data in large scales, we are facing the opportunities and challenges to analyze data with a large number of covariates per observation, the so-called high-dimensional problem. When this situation arises, variable selection is one of the most commonly used techniques, especially in radiological and genetic research, due to the nature of high-dimensional data extracted from imaging scans and gene sequencing. In the context of regression, when the number of covariates is greater than the sample size, the parameter estimation problem becomes ill posed, and variable selection is usually the first step for dimension reduction.

A good amount of work has recently been done for variable selection from both frequentist and Bayesian perspectives. On the frequentist side, extensive studies on variable selection have emerged ever since the appearance of least absolute shrinkage and selection operator (Lasso) [1]. Other penalization approaches for sparse model selection including smoothly clipped absolute deviation (SCAD) [2], minimum concave penalty (MCP) [3] and many variations have also been introduced. Most of these methods are first considered in the context of linear regression and then extended to generalized linear models. Because all the methods share the basic desire of shrinkage toward sparse models, it has been understood that most of these frequentist methods can be interpreted from a Bayesian perspective and many analogous Bayesian methods have also been proposed. See for example [4,5,6] that discuss the connection between penalized likelihood-based methods and Bayesian approaches. These Bayesian methods employed local priors, which still preserve positive values at null parameter values, to achieve desirable shrinkage.

In this paper, we are interested in nonlocal densities [7] that are identically zero whenever a model parameter is equal to its null value. Compared to local priors, nonlocal prior distributions have relatively appealing properties for Bayesian model selection. In particular, nonlocal priors discard spurious covariates faster as the sample size grows, while preserving exponential learning rates to detect nontrivial coefficients [7]. Johnson and Rossell [8] and Shin et al. [9] study the behavior of nonlocal densities for variable selection in a linear regression setting. When the number of covariates is much smaller than the sample size, [10] establish the posterior convergence rate for nonlocal priors in a logistic regression model and suggest a Metropolis–Hastings algorithm for computation.

To the best of our knowledge, a rigorous investigation of high-dimensional posterior consistency properties for nonlocal priors has not been undertaken in the context of generalized linear regression. Although [11] investigated the model selection consistency of nonlocal priors in generalized linear models, they assumed a fixed dimension *p*. Motivated by this gap, our first goal was to examine the model selection property for nonlocal priors, particularly, the product moment (pMOM) prior [8] in a high-dimensional generalized linear model. It is known that the computation problem can arise for Bayesian approaches due to the non-conjugate structure in generalized linear regression. Hence, our second goal was to develop efficient algorithms for exploring the massive posterior space. These were challenging goals of course, as the posterior distributions are not available in closed form for this type of nonlocal priors.

As the main contributions of this paper, we first establish model selection consistency for generalized linear models with pMOM prior on regression coefficients (Theorems 1–3) when the number of covariates grows at a sub-exponential rate of the sample size. Next, *n* terms of computation, we first obtain the posteriors via Laplace approximation and then implement an efficient shotgun stochastic search (SSS) algorithm for exploring the sparsity pattern of the regression coefficients. In particular, the SSS-based methods have been shown to significantly reduce the computational time compared with standard Markov chain Monte Carlo (MCMC) algorithms in various settings [9,12,13]. We demonstrate that our model can outperform existing state-of-the-art methods including both penalized likelihood and Bayesian approaches in different settings. Finally, the proposed method is applied to a functional Magnetic Resonance Imaging (fMRI) data set for identifying alternative brain activities and for predicting Parkinson’s disease.

The rest of paper is organized as follows. Section 2 provides background material regarding generalized linear models and revisits the pMOM distribution. We detail strong selection consistency results in Section 3, and proofs are provided in the Appendix A. The posterior computation algorithm is described in Section 4, and we show the performance of the proposed method and compare it with other competitors through simulation studies in Section 5. In Section 6, we conduct a real data analysis for predicting Parkinson’s disease and show our method yields better prediction performance compared with other contenders. To conclude our paper, a discussion is given in Section 7.

## 2. Preliminaries

### 2.1. Model Specification for Logistic Regression

We first describe the framework for Bayesian variable selection in logistic regression followed by our hierarchical model specification. Let $y\in {\left\{0,1\right\}}^{n}$ be the binary response vector and $X=\left(x_{ij}\right)\in {\mathbb{R}}^{n\times p}$ be the design matrix. Without loss of generality, we assume that the columns of X are standardized to have zero mean and unit variance. Let $x_{i}\in {\mathbb{R}}^{p}$ denote the *i*th row vector of X that contains the covariates for the *i*th subject. Let β be the p×1 vector of regression coefficients. We first consider the following standard logistic regression model:

(1) $$\begin{array}{c} \mathbb{P}\left(y_{i}=1|x_{i},\beta \right)=\frac{\exp\left(x_{i}^{⊤}\beta \right)}{1+\exp\left(x_{i}^{⊤}\beta \right)},\;i=1,2,\ldots ,n, \end{array}$$

We will work in a scenario where the dimension of predictors, *p* grows with the sample size *n*. Thus, we consider the number of predictors is function of *n*, i.e., $p=p_{n}$, but we denote it as *p* for notational simplicity.

Our goal is variable selection, i.e., the correct identification of all non-zero regression coefficients. In light of that, we denote a model by $k=\left\{k_{1},k_{2},\ldots ,k_{\left|k\right|}\right\}\subseteq \left[p\right]=:\left\{1,2,\ldots ,p\right\}$ if and only if all the nonzero elements of β are $\beta_{k_{1}},\beta_{k_{2}},\ldots ,\beta_{k_{\left|k\right|}}$ and denote $\beta_{k}={\left(\beta_{k_{1}},\beta_{k_{2}},\ldots ,\beta_{k_{\left|k\right|}}\right)}^{⊤}$, where |k| is the cardinality of k. For any m×p matrix *A*, let $A_{k}\in {\mathbb{R}}^{m\times |k|}$ denote the submatrix of A containing the columns of A indexed by model k. In particular, for 1≤i≤n, we denote $x_{ik}$ as the subvector of $x_{i}$ containing the entries of $x_{i}$ corresponding to model k.

The class of pMOM densities [8] can be used for model selection through the following hierarchical model

(2) $$\begin{array}{c} \pi \left(\beta_{k}|\tau ,k\right)=d_{k}{\left(2\pi \right)}^{-\frac{\left|k\right|}{2}}{\left(\tau \right)}^{-r|k|-\frac{\left|k\right|}{2}}{\left|U_{k}\right|}^{\frac{1}{2}}\exp\left(-\frac{{\beta_{k}}^{⊤}U_{k}\beta_{k}}{2\tau }\right)\prod_{i=1}^{\left|k\right|}\beta_{k_{i}}^{2r}, \end{array}$$

(3) $$\begin{array}{c} \pi \left(k\right)\propto I(|k|\leq m_{n}). \end{array}$$

Here U is a p×p nonsingular matrix, *r* is a positive integer referred to as the order of the density and $d_{k}$ is the normalizing constant independent of the positive constant τ. Please note that prior (2) is obtained as the product of the density of multivariate normal distribution and even powers of parameters, $\prod_{i=1}^{\left|k\right|}\beta_{k_{i}}^{2r}$. This results in $\pi (\beta_{k}|\tau ,k)=0$ at $\beta_{k}=0$, which is desirable because (2) is a prior for the nonzero elements of β. Some standard regularity assumptions on the hyperparameters will be provided later in Section 3. In (3), $m_{n}\in \left[p\right]$ is a positive integer restricting the size of the largest model, and a uniform prior is placed on the model space restricting our analysis to models having size less than or equal to $m_{n}$. Similar structure has also been considered in [5,9,14]. An alternative is to use a complexity prior [15] that takes the form of

$$\begin{array}{c} \pi \left(k\right)\propto c_{1}^{-|k|}p^{-c_{2}\left|k\right|}, \end{array}$$

for some positive constants $c_{1},c_{2}$. The essence is to force the estimated model to be sparse by penalizing dense models. As noted in [9], the model selection consistency result based on the nonlocal priors derives strength directly from the marginal likelihood and does not require strong penalty over model size. This is indeed reflected in the simulation studies in [14], where the authors compare the model selection performance under uniform prior and complexity prior. The result under uniform prior is much better than that under complexity prior, as the complexity prior always tends to prefer the sparse models.

By the hierarchical model (1) to (3) and Bayes’ rule, the resulting posterior probability for model k is denoted by,

(4) $$\begin{array}{c} \pi \left(k|y\right)=\frac{\pi (k)}{\pi (y)}m_{k}\left(y\right), \end{array}$$

where π(y) is the marginal density of y, and $m_{k}\left(y\right)$ is the marginal density of y under model k given by

(5) $$\begin{array}{cc} m_{k}\left(y\right) & =\int \exp\left\{L_{n}\left(\beta_{k}\right)\right\}\pi \left(\beta_{k}|k\right)d\beta_{k}\\ & =\int \exp\left\{L_{n}\left(\beta_{k}\right)\right\}d_{k}{\left(2\pi \right)}^{-|k|/2}{\left(\tau \right)}^{-r|k|-|k|/2}{\left|U_{k}\right|}^{\frac{1}{2}}\exp\left(-\frac{{\beta_{k}}^{⊤}U_{k}\beta_{k}}{2\tau }\right)\prod_{i=1}^{\left|k\right|}\beta_{k_{i}}^{2r}d\beta_{k}, \end{array}$$

where

(6) $$\begin{array}{c} L_{n}\left(\beta_{k}\right)=\log\left(\prod_{i=1}^{n}{\left\{\frac{\exp\left(x_{ik}^{⊤}\beta_{k}\right)}{1+\exp\left(x_{ik}^{⊤}\beta_{k}\right)}\right\}}^{y_{i}}{\left\{\frac{1}{1+\exp\left(x_{ik}^{⊤}\beta_{k}\right)}\right\}}^{1-y_{i}}\right) \end{array}$$

is the log likelihood function. In particular, these posterior probabilities can be used to select a model by computing the posterior mode defined by

(7) $$\hat{k}=arg\;max_{k}\pi \left(k|y\right).$$

Of course, the closed form of these posterior probabilities cannot be obtained due to not only the nature of logistic regression but also the structure of nonlocal prior. Therefore, special efforts need to be devoted to both consistency analysis and computational strategy as we shall see in the following sections.

### 2.2. Extension to Generalized Linear Model

We can easily extend our previous discussion on logistic regression to a generalized linear model (GLM) [16]. Given predictors $x_{i}$ and an outcome $y_{i}$ for 1≤i≤n, a probability density function (or probability mass function) of a generalized linear model has the following form of the exponential family

$$p\left(y_{i}|\theta \right)=\exp\left\{a\left(\theta \right)y_{i}+b\left(\theta \right)+c\left(y_{i}\right)\right\},$$

in which a(·) is a continuously differentiable function with respect to θ with nonzero derivative, b(·) is also a continuously differentiable function of θ, c(·) is some constant function of *y*, and θ is also known as the natural parameter that relates the response to the predictors through the linear function $\theta_{i}=\theta_{i}\left(\beta \right)=x_{i}^{⊤}\beta$. The mean function is $\mu =E\left(y_{i}|x_{i}\right)=-b^{\prime }\left(\theta_{i}\right)/a^{\prime }\left(\theta_{i}\right)≜\phi \left(\theta_{i}\right)$, where ϕ(·) is the inverse of some chosen link function.

The class of pMOM densities specified in (2) can still be used for model selection in this generalized setting by noting that the log likelihood function in (5) and (6) now takes the general form of

(8) $$\begin{array}{c} L_{n}\left(\beta_{k}\right)=\sum_{i=1}^{n}\left\{a\left(\theta_{i}\left(\beta_{k}\right)\right)y_{i}+b\left(\theta_{i}\left(\beta_{k}\right)\right)+c\left(y_{i}\right)\right\}. \end{array}$$

After obtaining the posterior probabilities in (4) with the log likelihood substituted as (8), we can select a model by computing the posterior mode. In Section 4, we will adopt some search algorithm that use these posterior probabilities to target the mode in a more efficient way.

## 3. Main Results

In this section, we show that the proposed Bayesian model enjoys desirable theoretical properties. Let t⊆[p] be the true model, which means that the nonzero locations of the true coefficient vector are t=(j,j∈t). We consider t to be a fixed vector. Let $\beta_{0}\in {\mathbb{R}}^{p}$ be the true coefficient vector and $\beta_{0,t}\in {\mathbb{R}}^{\left|t\right|}$ be the vector of the true nonzero coefficients. In the following analysis, we will focus on logistic regression, but our argument can be easily extended to any other GLM as well. In particular,

$$\begin{array}{ccc} H_{n}\left(\beta_{k}\right) & = & -\frac{\partial^{2}L_{n}\left(\beta_{k}\right)}{\partial \beta_{k}\partial {\beta_{k}}^{⊤}}\;\;=\;\;\sum_{i=1}^{n}\sigma_{i}^{2}\left(\beta_{k}\right)x_{i}x_{i}^{⊤}\;\;=\;\;{X_{k}}^{⊤}\Sigma \left(\beta_{k}\right)X_{k} \end{array}$$

as the negative Hessian of $L_{n}\left(\beta_{k}\right)$, where $\Sigma \left(\beta_{k}\right)\equiv \Sigma_{k}=diag\left(\sigma_{1}^{2}\left(\beta_{k}\right),\ldots ,\sigma_{n}^{2}\left(\beta_{k}\right)\right)$, $\sigma_{i}^{2}\left(\beta_{k}\right)=\mu_{i}\left(\beta_{k}\right)\left(1-\mu_{i}\left(\beta_{k}\right)\right)$ and

$$\begin{array}{ccc} \mu_{i}\left(\beta_{k}\right) & = & \frac{\exp\left(x_{ik}^{⊤}\beta_{k}\right)}{1+\exp\left(x_{ik}^{⊤}\beta_{k}\right)}. \end{array}$$

In the rest of the paper, we denote $\Sigma =\Sigma (\beta_{t})$ and $\sigma_{i}^{2}=\sigma_{i}^{2}\left(\beta_{t}\right)$ for simplicity.

Before we establish our main results, the following notations are needed for stating our assumptions. For any a,b∈ℝ, a∨b and a∧b mean the maximum and minimum of *a* and *b*, respectively. For any sequences $a_{n}$ and $b_{n}$, we denote $a_{n}≲b_{n}$, or equivalently $a_{n}=O\left(b_{n}\right)$, if there exists a constant C>0 such that $|a_{n}\left|\leq C\right|b_{n}|$ for all large *n*. We denote $a_{n}\ll b_{n}$, or equivalently $a_{n}=o\left(b_{n}\right)$, if $a_{n}/b_{n}⟶0$ as n→∞. Without loss of generality, if $a_{n}\geq b_{n}>0$ and there exist constants $C_{1}>C_{2}>0$ such that $C_{2}<b_{n}/a_{n}\leq a_{n}/b_{n}<C_{1}$, we denote $a_{n}∼b_{n}$. For a given vector $v={\left(v_{1},\ldots ,v_{p}\right)}^{⊤}\in {\mathbb{R}}^{p}$, the vector $\ell_{2}$-norm is denoted as ${\left\|v\right\|}_{2}={\left(\sum_{j=1}^{p}v_{j}^{2}\right)}^{1/2}$. For any real symmetric matrix A, $\lambda_{\max}\left(A\right)$ and $\lambda_{\min}\left(A\right)$ are the maximum and minimum eigenvalue of A, respectively. To attain desirable asymptotic properties of our posterior, we assume the following conditions:

**Condition (A1)**$\log n≲\log p=o(n^{1/2})$ and $m_{n}=O\left({\left(n/\log p\right)}^{\frac{1-d^{\prime }}{2}}\wedge \log p\right)$ for some $0\leq d<\left(1+d\right)/2\leq d^{\prime }\leq 1$.

Condition (A1) ensures our proposed method can accommodate high dimensions where the number of predictors grows at a sub-exponential rate of *n*. Condition (A1) also specifies the parameter $m_{n}$ in the uniform prior (3) that restricts our analysis on a set of *reasonably large* models. Similar assumptions restricting the model size have been commonly assumed in the sparse estimation literature [4,5,9,17].

**Condition (A2)** For some constant C∈(0,∞) and $0\leq d<\left(1+d\right)/2\leq d^{\prime }\leq 1$,

$$\begin{array}{ccc} \max_{i,j}\left|x_{ij}\right| & \leq & C,\\ 0<\lambda \leq \min_{k:|k|\leq m_{n}+\left|t\right|}\lambda_{\min}\left(n^{-1}H_{n}\left(\beta_{0,k}\right)\right) & \leq & \Lambda_{m_{n}+\left|t\right|}\leq C^{2}{\left(\log p\right)}^{d}, \end{array}$$

and $\Lambda_{\zeta }=\max_{k:|k|\leq \zeta }\lambda_{\max}\left(n^{-1}X_{k}^{⊤}X_{k}\right)$ for any integer ζ>0. Furthermore, $\|\beta_{0,t}{\|}_{2}^{2}=O\left({\left(\log p\right)}^{d}\right)$.

Condition (A2) gives lower and upper bounds of $n^{-1}H_{n}\left(\beta_{0,k}\right)$ and $n^{-1}X_{k}^{⊤}X_{k}$, respectively, where k is a large model satisfying $\left|k\right|\leq m_{n}+\left|t\right|$. The lower bound condition can be regarded as a restricted eigenvalue condition for $\ell_{0}$-sparse vectors. Restricted eigenvalue conditions are routinely assumed in high-dimensional theory to guarantee some level of curvature of the objective function and are satisfied with high probability for sub-Gaussian design matrices [5]. Similar conditions have also been used in the linear regression literature [18,19,20]. The last assumption in Condition (A2) says that the magnitude of true signals is bounded above ${\left(\log p\right)}^{d}$ up to some constant, which allows the magnitude of signals to increase to infinity.

**Condition (A3)** For some constant $c_{0}>0$,

(9) $$\begin{array}{ccc} \min_{j\in t}\beta_{0,j}^{2} & \geq & c_{0}\left(\frac{\left|t\right|\Lambda_{\left|t\right|}\log p}{n}\vee \frac{1}{\log p}\right). \end{array}$$

Condition (A3) gives a lower bound for nonzero signals, which is called the *beta-min* condition. In general, this type of condition is necessary for catching every nonzero signal. Please note that due to Conditions (A1) and (A2), the right-hand side of (9) decreases to zero as n→∞. Thus, it allows the smallest nonzero coefficients to tend to zero as we observe more data.

**Condition (A4)** For some small constant δ>0, the hyperparameters τ and *r* satisfy

$$\begin{array}{c} \tau^{r+1/2}∼n^{-1/2}p^{2+\delta }. \end{array}$$

Condition (A4) suggests appropriate conditions for the hyperparameter τ in (2). A similar assumption has also been considered in [9]. The scale parameter τ in the nonlocal prior density reflects the dispersion of the nonlocal prior density around zero, and implicitly determines the size of the regression coefficients that will be shrunk to zero [8,9]. For the below theoretical results, we assume that U=I for simplicity, but our results are still valid for other choices of U as long as $\lambda_{\max}\left(U\right)=O\left(1\right)$ and $\lambda_{\min}\left(U\right)=O\left(1\right)$.

**Theorem** **1** (No super set). *Under conditions* (A1)*,* (A2) *and* (A4)*,*

$$\begin{array}{ccc} \pi \left(k⊋t|y\right) & \overset{P}{⟶} & 0,\;\;\;\mathrm{as}\;n\to \infty . \end{array}$$

Theorem 1 says that, asymptotically, our posterior will not overfit the model, i.e., not include unnecessarily many variables. Of course, it does not guarantee that the posterior will concentrate on the true model. To capture every significant variable, we require the magnitudes of nonzero entries in $\beta_{0,t}$ not to be too small. Theorem 2 shows that with an appropriate lower bound specified in Condition (A3), the true model t will be the mode of the posterior.

**Theorem** **2** (Posterior ratio consistency). *Under conditions* (A1)*–*(A4) *with $c_{0}={\left\{\left(1-\epsilon_{0}\right)\lambda \right\}}^{-1}\left\{2\left(3+\delta \right)+5{\left\{\left(1-\epsilon_{0}\right)\lambda \right\}}^{-1}\right\}$ for some small constant $\epsilon_{0}>0$,*

$$\begin{array}{ccc} \max_{k\neq t}\frac{\pi \left(k|y\right)}{\pi \left(t|y\right)} & \overset{P}{⟶} & 0,\;\;\;\mathrm{as}\;n\to \infty . \end{array}$$

Posterior ratio consistency is a useful property especially when we are interested in the point estimation with the posterior mode, but does not provide how large probability the posterior puts on the true model. In the following theorem, we state that our posterior achieves *strong selection consistency*. By strong selection consistency, we mean that the posterior probability assigned to the true model *t* converges to 1. Since strong selection consistency implies posterior ratio consistency, it requires a slightly stronger condition on the lower bound for the magnitudes of nonzero entries in $\beta_{0,t}$, i.e., a larger value of $c_{0}$, compared to that in Theorem 2.

**Theorem** **3** (Strong selection consistency). *Under conditions* (A1)*–*(A4) *with $c_{0}={\left\{\left(1-\epsilon_{0}\right)\lambda \right\}}^{-1}\left\{2\left(9+2\delta \right)+5{\left\{\left(1-\epsilon_{0}\right)\lambda \right\}}^{-1}\right\}$ for some small constant $\epsilon_{0}>0$, the following holds:*

$$\begin{array}{ccc} \pi \left(t|y\right) & \overset{P}{⟶} & 1,\;\;\;\mathrm{as}\;n\to \infty . \end{array}$$

## 4. Computational Strategy

In this section, we describe how to approximate the marginal density of the data and to conduct the model selection procedure. The integral formulation in (4) leads to the posterior probabilities not available in closed form. Hence, we use Laplace approximation to compute $m_{k}\left(y\right)$ and π(k|y). A similar approach to compute posterior probabilities has been used in [8,9,10].

Please note that for any model k, when $U_{k}=I_{k}$, the normalization constant $d_{k}$ in (2) is given by $d_{k}={\left\{(2r-1)!!\right\}}^{-|k|}$. Let

$$\begin{array}{ccc} f(\beta_{k}) & = & \log\left(\exp\left\{L_{n}\left(\beta_{k}\right)\right\}\pi \left(\beta_{k}|k\right)\right)\\ & = & \sum_{i=1}^{n}\left\{y_{i}x_{ik}^{⊤}\beta_{k}-\log\left(1+\exp(x_{ik}^{⊤}\beta_{k})\right)\right\}-\left|k\right|\log\left((2r-1)!!\right)-\frac{\left|k\right|}{2}\log\left(2\pi \right)-\left(r|k|+\frac{\left|k\right|}{2}\right)\log\tau \\ & - & \frac{\beta_{k}^{⊤}\beta_{k}}{2\tau }+\sum_{i=1}^{\left|k\right|}2r\log\left(|\beta_{k_{i}}|\right). \end{array}$$

For any model k, the Laplace approximation of $m_{k}\left(y\right)$ is given by

(10) $$\begin{array}{c} {\left(2\pi \right)}^{\frac{\left|k\right|}{2}}\exp\left\{f({\hat{\beta }}_{k})\right\}{\left|V\left({\hat{\beta }}_{k}\right)\right|}^{-\frac{1}{2}}, \end{array}$$

where ${\hat{\beta }}_{k}=arg\;max_{\beta_{k}}f\left(\beta_{k}\right)$ obtained via the optimization function optim in R using a quasi-Newton method and $V({\hat{\beta }}_{k})$ is a |k|×|k| symmetric matrix which can be calculated as:

$$\begin{array}{c} -\sum_{i=1}^{n}\frac{x_{ik}x_{ik}^{⊤}\exp\left(x_{ik}^{⊤}\beta_{k}\right)}{{\left\{1+\exp(x_{ik}^{⊤}\beta_{k})\right\}}^{2}}-\frac{1}{\tau }I_{k}-diag\left(\frac{2r}{\beta_{k_{1}}^{2}},\ldots ,\frac{2r}{\beta_{k_{\left|k\right|}}^{2}}\right). \end{array}$$

The above Laplace approximation can be used to compute the log of the posterior probability ratio between any given model k and true model t, and select a model k with the highest probability.

We then adopt the shotgun stochastic search (SSS) algorithm [9,12] to efficiently navigate through the massive model space and identify the global maxima. Using the Laplace approximations of the marginal probabilities in (11), the SSS method aims at exploring “interesting” regions of the resulting high-dimensional model spaces and quickly identifies regions of high posterior probability over models. Let $nbd\left(k\right)=\left\{\Gamma^{+},\Gamma^{-},\Gamma^{0}\right\}$ containing all the neighbors of model k, in which $\Gamma^{+}=\left\{k\;\cup \{j\}:j\notin k\right\}$, $\Gamma^{-}=\left\{k\setminus \{j\}:j\in k\right\}$ and $\Gamma^{0}=\left\{k\setminus \{j\}\cup \{l\}:j\in k,l\notin k\right\}$. The SSS procedure is described in Algorithm 1.
**Algorithm 1** Shotgun Stochastic Search (SSS)Choose an initial model $k^{\left(1\right)}$ **for**
i=1 to i=N−1
**do**    Compute π(k|y) for all $k\in \mathrm{nbd}\left(k^{\left(i\right)}\right)$    Sample $k^{+}$, $k^{-}$ and $k^{0}$, from $\Gamma^{+}$, $\Gamma^{-}$ and $\Gamma^{0}$ with probabilities proportional to π(k|y)    Sample the next model $k^{\left(i+1\right)}$ from $\left\{k^{+},k^{-},k^{0}\right\}$ with probability proportional to    $\left\{\pi \left(k^{+}|y\right),\pi \left(k^{-}|y\right),\pi \left(k^{0}|y\right)\right\}$ **end for**

## 5. Simulation Studies

In this section, we demonstrate the performance of the proposed method in various settings. Let X be the design matrix whose first |t| columns correspond to the active covariates for which we have nonzero coefficients, while the rest correspond to the inactive ones with zero coefficients. In all the simulation settings, we generate $x_{i}\overset{i.i.d.}{∼}N_{p}\left(0,\Sigma \right)$ for i=1,…,n under the following two different cases of Σ:

- Case 1: Isotropic design, where $\Sigma =I_{p}$, i.e., no correlation imposed between different covariates.
- Case 2: Autoregressive correlated design, where $\Sigma_{ij}=0.3^{\left|i-j\right|},$ for all 1≤i≤j≤p. The correlations among different covariates are set to different values.

Following the simulation settings in [9,10], we consider the following two designs, each with the same sample size n=100 and number of predictors being either p=100 or 150:

- Design 1 (Dense model): The number of predictors p=100 and |t|=8.
- Design 2 (High-dimensional): The number of predictors p=150 and |t|=4.

We investigate the following two settings for the true coefficient vector $\beta_{0,t}$ to include different combinations of small and large signals.

- Setting 1: All the entries of $\beta_{0,t}$ are set to 3.
- Setting 2: All the entries of $\beta_{0,t}$ are generated from Unif(1.5,3).

Finally, for given X and 1≤i≤n, we sample $y_{i}$ from the following logistic model as in (1)

$$\begin{array}{c} \mathbb{P}\left(y_{i}=1|x_{i},\beta_{0}\right)=\frac{\exp\left(x_{i}^{⊤}\beta_{0}\right)}{1+\exp\left(x_{i}^{⊤}\beta_{0}\right)}. \end{array}$$

We will refer to our proposed method as “nonlocal” and its performance will then be compared with other existing methods including Spike and Slab prior-based model selection [21], empirical Bayesian LASSO (EBLasso) [22], Lasso [23] and SCAD [24]. The tuning parameters in the regularization approaches are chosen by 5-fold cross-validation. Spike and slab prior method is implemented via the BoomSpikeSlab package in R. For the nonlocal prior, the hyperparameters are set at U=I, r=1 and we tune $\tau =10^{-i}n^{-1/2}p^{2+0.05}$ for four different values of i=0,1,2,3. We choose the optimal τ by the mean squared prediction error through 5-fold cross-validation. Please note that this implies that τ is data-dependent and the resulting procedure is similar to an empirical-Bayesian approach in the high-dimensional Bayesian literature given the prior knowledge about the sparse true model [13]. For the SSS procedure, the initial model was set by randomly taking three coefficients to be active and the remaining to be inactive. The detailed steps for our method are coded in R and publicly available at https://github.com/xuan-cao/Nonlocal-Logistic-Selection. In particular, the stochastic search is implemented via the SSS function in the R package BayesS5.

To evaluate the performance of variable selection, the precision, sensitivity, specificity, Matthews correlation coefficient (MCC) [25] and mean-squared prediction error (MSPE) are reported at Table 1, Table 2, Table 3 and Table 4, where each simulation setting is repeated for 20 times. The criteria are defined as

$$\begin{array}{c} \mathrm{Precision}=\frac{TP}{TP+FP},\;\mathrm{Sensitivitiy}=\frac{TP}{TP+FN},\;\mathrm{Specificity}=\frac{TN}{TN+FP},\\ \mathrm{MCC}=\frac{TP\times TN-FP\times FN}{\sqrt{\left(TP+FP)(TP+FN)(TN+FP)(TN+FN\right)}},\;\mathrm{MSPE}=\frac{1}{n_{\mathrm{test}}}\sum_{i=1}^{n_{\mathrm{test}}}{\left({\hat{y}}_{i}-y_{\mathrm{test},i}\right)}^{2}, \end{array}$$

where *TP*, *TN*, *FP* and *FN* are true positive, true negative, false positive and false negative, respectively. Here we denote ${\hat{y}}_{i}=x_{i}^{⊤}\hat{\beta }$, where $\hat{\beta }$ is the estimated coefficient based on each method. For Bayesian methods, the usual GLM estimates based on the selected support are used as $\hat{\beta }$. We generated test samples $y_{\mathrm{test},1},\ldots ,y_{\mathrm{test},n_{\mathrm{test}}}$ with $n_{\mathrm{test}}=50$ to calculate the MSPE.

Based on the above simulation results, we notice that under the first isotropic covariance case, the nonlocal-based approach overall works better than other methods especially in the strong signal setting (i.e., Setting 1), where our method is able to consistently achieve perfect estimation accuracy. This is because as signal strength gets stronger, the consistency conditions of our method are easier to satisfy which leads to better performance. When the covariance is autoregressive, our method suffers from lower sensitivity compared with the frequentist approaches in high-dimensional design (Table 4), but still has higher precision, specificity and MCC. The poor precision of the regularization methods has also been discussed in previous literature in the sense that selection of the regularization parameter using cross-validation is optimal with respect to prediction but tends to include too many noise predictors [26]. Again we observe under the autoregressive design, the performance of our method improves as the true signals strengthen. To sum up, the above simulation studies indicate that the proposed method can perform well under a variety of configurations with different data generation mechanisms.

## 6. Application to fMRI Data Analysis

In this section, we apply the proposed model selection method to an fMRI data set for identifying aberrant functional brain activities to aid the diagnosis of Parkinson’s Disease (PD) [27]. Data consists of 70 PD patients and 50 healthy controls (HC). All the demographic characteristics and clinical symptom ratings have been collected before MRI scanning. In particular, we adopt the mini-mental state examination (MMSE) for cognitive evaluation and the Hamilton Depression Scale (HAMD) for measuring the severity of depression.

### 6.1. Image Feature Extraction

Functional imaging data for all subjects are collected and retrieved from the archive by neuroradiologists. Image preprocessing procedure is carried out via Statistical Parametric Mapping (SPM12) operated on the Matlab platform. For each subject, we first discard the first 5 time points for signal equilibrium and the remaining 135 images underwent slice-timing and head motion corrections. Four subjects with more than 2.5 mm maximum displacement in any of the three dimensions or $2.5^{\circ }$ of any angular motion are removed. The functional images are spatially normalized to the Montreal Neurological Institute space with 3×3×3 mm${}^{3}$ cubic voxels and smoothed with a 4 mm full width at half maximum (FWHM) Gaussian kernel. We further regress out nuisance covariates and applied temporal filter (0.01 Hz <f< 0.08 Hz) to diminish high-frequency noise.

Zang et al. [28] proposed the method of Regional Homogeneity (ReHo) to analyze characteristics of regional brain activity and to reflect the temporal homogeneity of neural activity. Since some preprocessing methods especially spatial smoothing fMRI time series may significantly change the ReHo magnitudes [29], preprocessed fMRI data without the spatial smoothing step are used for calculating ReHo. In particular, we focus on the mReHo maps obtained by dividing the mean ReHo of the whole brain within each voxel in the ReHo map. We further segment the mReHo maps and extract all the 112 ROI signals based on the Harvard-Oxford atlas (HOA) using the Resting-State fMRI Data Analysis Toolkit.

Slow fluctuations in activity are fundamental features of the resting brain for determining correlated activity between brain regions and resting state networks. The relative magnitude of these fluctuations can discriminate between brain regions and subjects. Amplitude of Low Frequency Fluctuations (ALFF) [30] are related measures that quantify the amplitude of these low frequency oscillations. Leveraging the preprocessed data, we retain the standardized mALFF maps after dividing the ALFF of each voxel by the global mean ALFF. Using the HOA, we again obtain 112 mALFF values via extracting the ROI signals based on the mALFF maps. Voxel-Mirrored Homotopic Connectivity (VMHC) quantifies functional homotopy by providing a voxel-wise measure of connectivity between hemispheres [31]. By segmenting the VMHC maps according to HOA, we also extract 112 VHMC values.

### 6.2. Results

Our candidate features consist of 336 radiomic variables along with all the clinical characteristics. We now consider a standard logistic regression model with the binary disease indicator as the outcome and all the radiomic variables together with five clinical factors as predictors. Various models including the proposed and other competing methods will then be implemented for classifying subjects based on these extracted features. The dataset is randomly divided into a training set (80%) and a testing set (20%) while maintaining the PD:HC ratio in both sets. For Bayesian methods, we first obtain the identified variables, and then evaluate the testing set performance using standard GLM estimates based on the selected features. The penalty parameters in all frequentist methods are tuned via 5-fold cross validation in the training set. The hyperparameters for the proposed method are set as in simulation studies.

**Figure 1:**
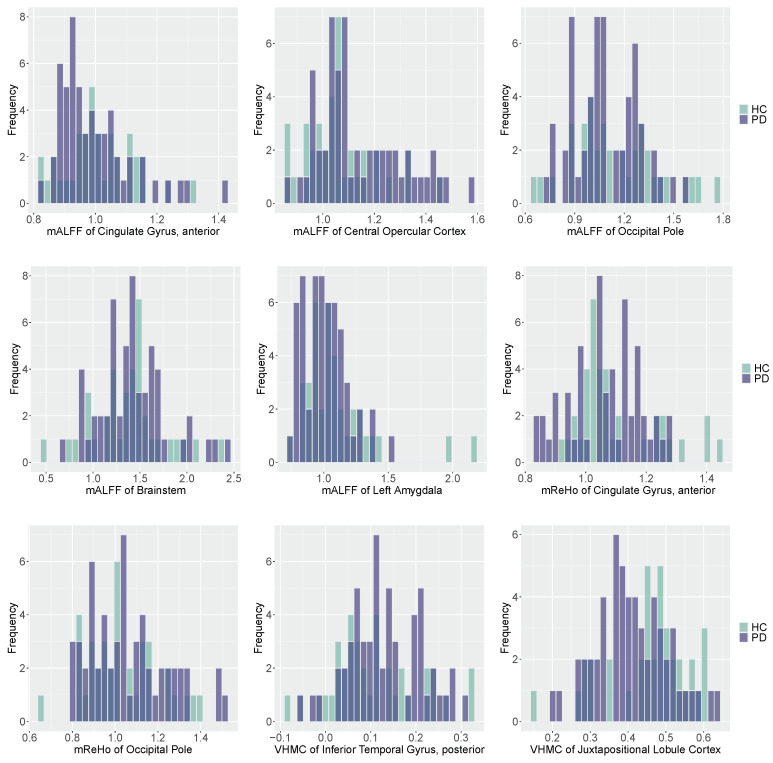
Histograms of selected radiomic features for PD and HC subjects with darker color representing overlapping values. Purple: PD group; Green: HC group.

The HAMD score and nine radiomic features including five mALFFs, two ReHos, two VHMCs are selected by the SSS procedure under pMOM prior. In Figure 1, we plot the histograms of selected radiomic features with different colors representing different groups. The predictive performance of various methods in the test set is summarized in Table 5. We can tell from Table 5 that the nonlocal prior-based approach has overall better prediction performance compared with other methods. Our nonlocal approach has higher precision and specificity compared with all the other methods, but yields a lower sensitivity than the frequentist approaches. Based on the most comprehensive measure MCC, our method outperforms all the other methods.

## 7. Conclusions

In this paper, we propose a Bayesian hierarchical model with a pMOM prior specification over regression coefficients to perform variable selection in high-dimensional generalized linear models. The model selection consistency of our method is established under mild conditions and the shotgun stochastic search algorithm can be used for the implementation of our proposed approach. Our simulation and real data studies indicate that the proposed method has better performance for variable selection compared to a variety of state-of-the-art competing methods. In the fMRI data analysis, our method is able to identify abnormal functional brain activities for PD that occur in the regions of interest including cingulate gyrus, central opercular cortex, occipital pole, brainstem, left amygdala, occipital pole, inferior temporal gyrus, and juxtapositional lobule cortex. These findings suggest disease-related alterations of functional activities that provide physicians sufficient information to get involved with early diagnosis and treatment. Our findings are also coherent with the alternative functional features in cortical regions, brainstem, and limbic regions discovered in previous studies [32,33,34,35].

Our fMRI study certainly has limitations. First, we would like to note that fMRI data are typically treated as spatio-temporal objects and a generalized linear model with spatially varying coefficients can be implemented for brain decoding [36]. However, in our application, for each subject, a total of 135 fMRI scans were obtained, each with the dimension of 64×64×31. If we take each voxel as a covariate to perform the whole-brain functional analysis, it would be computationally challenging and impractical given the extremely high dimension. Hence, we adopt the radiomics approach to extract three different types of features that can summarize the functional activity of the brain, and take these radiomic features as covariates in our generalized linear model. For future studies, we will focus on several regions of interest rather than the entire brain and take the spatio-temporal dependency among voxels into consideration.

Second, although ReHo, ALFF, and VHMC are different types of radiomic features that quantify the functional activity of the brain, it is definitely possible that in some regions, three measures are highly correlated with each other. Our current theoretical and computational strategy can accommodate a reasonable amount of correlations among covariates, but might not work in the presence of high correlation structure. For future studies, we will first carefully examine the potential correlations among features and might only retain one feature for each region if significant correlations are detected.

One possible extension of our methodology is to address the potential misspecification of the hyperparameter τ. The scale parameter τ is of particular importance in the sense that it can reflect the dispersion of the nonlocal density around zero, and implicitly determine the size of the regression coefficients that will be shrunk to zero [8]. Cao et al. [14] investigated the model selection consistency for the hyper-pMOM priors in linear regression setting, where an additional inverse-gamma prior is placed over τ. Wu et al. [11] proved the model selection consistency using hyper-pMOM prior in generalized linear models, but assumed a fixed dimension *p*. For future study, we will consider this fully Bayesian approach to carefully examine the theoretical and empirical properties for such hyper-pMOM prior in the context of high-dimensional generalized linear regression. We can also extend our method to develop a Bayesian approach for growth models in the context of non-linear regression [37], where the log-transformation is typically used to recover the additive structure. However, then the model does not fall into the category of GLMs, which is beyond the current setting in this paper. Therefore, we leave it as a future work.

## Figures and Tables

**Table 1:** The summary statistics for Design 1 (Dense model design) are represented for each setting of the true regression coefficients under the first isotropic covariance case. Different setting means different choice of the true coefficient $\beta_{0}$.

	**Setting 1**				
	**Precision**	**Sensitivity**	**Specificity**	**MCC**	**MSPE**
Nonlocal	1	1	1	1	0.02
Spike and Slab	1	0.38	1	0.60	0.21
Lasso	0.67	1	0.96	0.80	0.17
EBLasso	1	0.38	1	0.60	0.22
SCAD	0.57	1	0.93	0.73	0.14
	**Setting 2**				
	**Precision**	**Sensitivity**	**Specificity**	**MCC**	**MSPE**
Nonlocal	0.73	1	0.97	0.84	0.18
Spike and Slab	1	0.13	1	0.34	0.23
Lasso	0.54	0.88	0.93	0.65	0.15
EBLasso	1	0.63	1	0.78	0.22
SCAD	0.47	0.88	0.91	0.60	0.13

**Table 2:** The summary statistics for Design 1 (Dense model design) are represented for each setting of the true regression coefficients under the second autoregressive covariance case. Different setting means different choice of the true coefficient $\beta_{0}$.

	**Setting 1**				
	**Precision**	**Sensitivity**	**Specificity**	**MCC**	**MSPE**
Nonlocal	0.89	1	0.99	0.94	0.13
Spike and Slab	0.71	0.63	0.98	0.64	0.20
Lasso	0.70	0.88	0.98	0.76	0.16
EBLasso	1	0.50	1	0.69	0.23
SCAD	0.67	0.75	0.97	0.68	0.17
	**Setting 2**				
	**Precision**	**Sensitivity**	**Specificity**	**MCC**	**MSPE**
Nonlocal	0.88	0.88	0.99	0.86	0.14
Spike and Slab	0.83	0.63	0.99	0.70	0.13
Lasso	0.63	0.88	0.96	0.72	0.14
EBLasso	1	0.38	1	0.60	0.22
SCAD	0.47	0.88	0.91	0.60	0.13

**Table 3:** The summary statistics for Design 2 (High-dimensional design) are represented for each setting of the true regression coefficients under the first isotropic covariance case. Different setting means different choice of the true coefficient $\beta_{0}$.

	**Setting 1**				
	**Precision**	**Sensitivity**	**Specificity**	**MCC**	**MSPE**
Nonlocal	1	1	1	1	0.08
Spike and Slab	0.75	0.75	0.99	0.74	0.09
Lasso	0.80	1	0.99	0.89	0.14
EBLasso	1	0.75	1	0.86	0.21
SCAD	0.67	1	0.99	0.81	0.12
	**Setting 2**				
	**Precision**	**Sensitivity**	**Specificity**	**MCC**	**MSPE**
Nonlocal	1	1	1	1	0.10
Spike and Slab	0.75	0.75	0.99	0.74	0.11
Lasso	0.67	1	0.99	0.81	0.14
EBLasso	1	0.75	1	0.86	0.23
SCAD	0.44	1	0.97	0.66	0.12

**Table 4:** The summary statistics for Design 2 (High-dimensional design) are represented for each setting of the true regression coefficients under the second autoregressive covariance case. Different setting means different choice of the true coefficient $\beta_{0}$.

	**Setting 1**				
	**Precision**	**Sensitivity**	**Specificity**	**MCC**	**MSPE**
Nonlocal	1	0.75	1	0.86	0.11
Spike and Slab	1	0.50	1	0.71	0.10
Lasso	0.57	1	0.98	0.75	0.10
EBLasso	1	0.50	1	0.70	0.18
SCAD	0.44	1	0.97	0.66	0.12
	**Setting 2**				
	**Precision**	**Sensitivity**	**Specificity**	**MCC**	**MSPE**
Nonlocal	1	0.75	1	0.86	0.15
Spike and Slab	0.50	0.50	0.99	0.49	0.14
Lasso	0.44	1	0.97	0.66	0.13
EBLasso	1	0.50	1	0.70	0.21
SCAD	0.40	1	0.96	0.62	0.14

**Table 5:** The summary statistics for prediction performance on the testing set for all methods.

	Precision	Sensitivity	Specificity	MCC	MSPE
Nonlocal	0.77	0.83	0.73	0.56	0.21
Spike and Slab	0.53	0.75	0.27	0.40	0.29
Lasso	0.67	1	0.45	0.55	0.18
EBLasso	0.57	1	0.18	0.32	0.28
SCAD	0.58	1	0.37	0.41	0.19

## Abbreviations

The following abbreviations are used in this manuscript:

Lasso	least absolute shrinkage and selection operator
SCAD	smoothly clipped absolute deviation
MCP	minimum concave penalty
pMOM	product moment
SSS	shotgun stochastic search
MCMC	Markov chain Monte Carlo
fMRI	functional Magnetic Resonance Imaging
GLM	generalized linear model
EBLasso	empirical Bayesian LASSO
MCC	Matthews correlation coefficient
MSPE	mean-squared prediction error
PD	Parkinson’s Disease
HC	healthy controls
MMSE	mini-mental state examination
HAMD	Hamilton Depression Scale
SPM12	Statistical Parametric Mapping
FWHM	full width at half maximum
HOA	Harvard-Oxford atlas
ALFF	Amplitude of Low Frequency Fluctuations
VMHC	Voxel-Mirrored Homotopic Connectivity

## Appendix A

Throughout the Supplementary Material, we assume that for any

$$u\in \{u\in {\mathbb{R}}^{n}:u\;\mathrm{is}\;\mathrm{in}\;\mathrm{the}\;\mathrm{space}\;\mathrm{spanned}\;\mathrm{by}\;\mathrm{the}\;\mathrm{columns}\;\mathrm{of}\;\Sigma^{1/2}X_{k}\}$$

and any model $k\in \{k\subseteq \left[r\right]:|k|\leq m_{n}+|t|\}$, there exists $\delta^{*}>0$ such that

(A1) $$\begin{array}{ccc} 𝔼\left[\exp\left\{u^{⊤}\Sigma^{-1/2}\left(y-\mu \right)\right\}\right] & \leq & \exp\left\{\frac{\left(1+\delta^{*}\right)u^{⊤}u}{2}\right\}, \end{array}$$

for any $n\geq N(\delta^{*})$. However, as stated in [5], there always exists $\delta^{*}>0$ satisfying inequality (A1), so it is not really a restriction. Since we will focus on sufficiently large *n*, $\delta^{*}$ can be considered an arbitrarily small constant, so we can always assume that $\delta >\delta^{*}$.

**Proof of Theorem** **1.** Let $M_{1}=\{k:k⊋t,|k|\leq m_{n}\}$ and

$$\begin{array}{ccc} PR(k,t) & = & \frac{\pi (k|y)}{\pi (t|y)}, \end{array}$$

where t⊆[r] is the true model. We will show that

(A2) $$\begin{array}{ccc} \sum_{k:k\in M_{1}}PR\left(k,t\right) & \overset{P}{⟶} & 0\;\;\mathrm{as}\;n\to \infty . \end{array}$$

By Taylor’s expansion of $L_{n}\left(\beta_{k}\right)$ around ${\hat{\beta }}_{k}$, which is the MLE of $\beta_{k}$ under the model k, we have

$$\begin{array}{ccc} L_{n}\left(\beta_{k}\right) & = & L_{n}\left({\hat{\beta }}_{k}\right)-\frac{1}{2}{\left(\beta_{k}-{\hat{\beta }}_{k}\right)}^{⊤}H_{n}\left({\tilde{\beta }}_{k}\right)\left(\beta_{k}-{\hat{\beta }}_{k}\right) \end{array}$$

for some ${\tilde{\beta }}_{k}$ such that $\|{\tilde{\beta }}_{k}-{\hat{\beta }}_{k}{\|}_{2}\leq {\left\|\beta_{k}-{\hat{\beta }}_{k}\right\|}_{2}$. Furthermore, by Lemmas A.1 and A.3 in [5] and Condition (A2), with probability tending to 1,

$$\begin{array}{ccc} L_{n}\left(\beta_{k}\right)-L_{n}\left({\hat{\beta }}_{k}\right) & \leq & -\frac{1-\epsilon }{2}{\left(\beta_{k}-{\hat{\beta }}_{k}\right)}^{⊤}H_{n}\left(\beta_{0,k}\right)\left(\beta_{k}-{\hat{\beta }}_{k}\right) \end{array}$$

for any $k\in M_{1}$ and $\beta_{k}$ such that $\|\beta_{k}-\beta_{0,k}{\|}_{2}<c\sqrt{\left|k\right|\Lambda_{\left|k\right|}\log p/n}=:cw_{n}$, where $\epsilon =\epsilon_{n}:=c^{\prime }\sqrt{m_{n}^{2}\Lambda_{m_{n}}\log p/n}=o\left(1\right)$, for some constants $c,c^{\prime }>0$. Please note that for $\beta_{k}$ such that $\|\beta_{k}-{\hat{\beta }}_{k}{\|}_{2}=cw_{n}/2$,

$$\begin{array}{ccc} L_{n}\left(\beta_{k}\right)-L_{n}\left({\hat{\beta }}_{k}\right) & \leq & -\frac{1-\epsilon }{2}\;{\left\|\beta_{k}-{\hat{\beta }}_{k}\right\|}_{2}^{2}\;\lambda_{\min}\left\{H_{n}\left(\beta_{0,k}\right)\right\}\\ & \leq & -\frac{1-\epsilon }{2}\;\frac{c^{2}w_{n}^{2}}{4}n\lambda =-\frac{1-\epsilon }{8}c^{2}\lambda \left|k\right|\Lambda_{\left|k\right|}\log p\;\;⟶\;\;-\infty \;\;\mathrm{as}\;n\to \infty , \end{array}$$

where the second inequality holds due to Condition (A2). It also holds for any $\beta_{k}$ such that $\|\beta_{k}-{\hat{\beta }}_{k}{\|}_{2}>cw_{n}/2$ by concavity of $L_{n}\left(\cdot \right)$ and the fact that ${\hat{\beta }}_{k}$ maximizes $L_{n}\left(\beta_{k}\right)$. Define the set $B:=\left\{\beta_{k}:{\left\|\beta_{k}-{\hat{\beta }}_{k}\right\|}_{2}\leq cw_{n}/2\right\},$ then we have $B\subset \{\beta_{k}:\|\beta_{k}-\beta_{0,k}{\|}_{2}\leq cw_{n}\}$ for some large c>0 and any $k\in M_{1}$, with probability tending to 1.

(A3) $$\begin{array}{ccc} m_{k}\left(y\right) & = & \int \exp\left\{L_{n}\left(\beta_{k}\right)\right\}\pi \left(\beta_{k}|k\right)d\beta_{k}\\ & = & \int \exp\left\{L_{n}\left(\beta_{k}\right)\right\}d_{k}{\left(2\pi \right)}^{-|k|/2}{\left(\tau \right)}^{-r|k|-|k|/2}{\left|U_{k}\right|}^{\frac{1}{2}}\exp\left(-\frac{{\beta_{k}}^{⊤}U_{k}\beta_{k}}{2\tau }\right)\prod_{i=1}^{\left|k\right|}\beta_{k_{i}}^{2r}d\beta_{k}\\ & \leq & d_{k}{\left(2\pi \right)}^{-|k|/2}{\left(\tau \right)}^{-r|k|-|k|/2}{\left|U_{k}\right|}^{\frac{1}{2}}\exp\left\{L_{n}\left({\hat{\beta }}_{k}\right)\right\}\\ & & \times [\int_{B}\exp\left\{-\frac{1-\epsilon }{2}{\left(\beta_{k}-{\hat{\beta }}_{k}\right)}^{⊤}H_{n}\left(\beta_{0,k}\right)\left(\beta_{k}-{\hat{\beta }}_{k}\right)-\frac{{\beta_{k}}^{⊤}U_{k}\beta_{k}}{2\tau }\right\}\prod_{i=1}^{\left|k\right|}\beta_{k_{i}}^{2r}d\beta_{k}\\ & & \;+\exp\left(-\frac{1-\epsilon }{8}c^{2}\lambda \left|k\right|\Lambda_{\left|k\right|}\log p\right)\int_{B^{c}}\exp\left(-\frac{{\beta_{k}}^{⊤}U_{k}\beta_{k}}{2\tau }\right)\prod_{i=1}^{\left|k\right|}\beta_{k_{i}}^{2r}d\beta_{k}] \end{array}$$

Please note that for $A_{k}=\left(1-\epsilon \right)H_{n}\left(\beta_{0,k}\right)$ and ${\beta_{k}}^{*}={\left(A_{k}+U_{k}/\tau \right)}^{-1}A_{k}{\hat{\beta }}_{k}$, we have

$$\begin{array}{cc} & \int_{B}\exp\left\{-\frac{1-\epsilon }{2}{\left(\beta_{k}-{\hat{\beta }}_{k}\right)}^{⊤}H_{n}\left(\beta_{0,k}\right)\left(\beta_{k}-{\hat{\beta }}_{k}\right)-\frac{{\beta_{k}}^{⊤}U_{k}\beta_{k}}{2\tau }\right\}\prod_{i=1}^{\left|k\right|}\beta_{k_{i}}^{2r}d\beta_{k}\\ \leq & \int \exp\left\{-\frac{1}{2}{\left(\beta_{k}-\beta_{k}^{*}\right)}^{⊤}\left(A_{k}+U_{k}/\tau \right)\left(\beta_{k}-\beta_{k}^{*}\right)\right\}\prod_{i=1}^{\left|k\right|}\beta_{k_{i}}^{2r}d\beta_{k}\\ & \times \;\;\exp\left\{-\frac{1}{2}{\hat{\beta }}_{k}^{⊤}\left(A_{k}-A_{k}{\left(A_{k}+U_{k}/\tau \right)}^{-1}A_{k}\right){\hat{\beta }}_{k}\right\}\\ = & {\left(2\pi \right)}^{|k|/2}\det{\left(A_{k}+U_{k}/\tau \right)}^{-1/2}\;\exp\left\{-\frac{1}{2}{\hat{\beta }}_{k}^{⊤}\left(A_{k}-A_{k}{\left(A_{k}+U_{k}/\tau \right)}^{-1}A_{k}\right){\hat{\beta }}_{k}\right\}E_{k}\left(\prod_{i=1}^{\left|k\right|}\beta_{k_{i}}^{2r}\right), \end{array}$$

where $E_{k}\left(.\right)$ denotes the expectation with respect to a multivariate normal distribution with mean ${\beta_{k}}^{*}$ and covariance matrix ${\left(A_{k}+U_{k}/\tau \right)}^{-1}$. It follows from Lemma 6 in the supplementary material for [8] that

$$\begin{array}{ccc} E_{k}\left(\prod_{i=1}^{\left|k\right|}\beta_{k_{i}}^{2r}\right) & \leq & {\left(\frac{n\Lambda_{\left|k\right|}+\tau^{-1}}{n\lambda +\tau^{-1}}\right)}^{|k|/2}{\left\{\frac{4V}{\left|k\right|}+\frac{4{\left[(2r-1)!!\right]}^{\frac{1}{r}}}{n(\lambda +\tau^{-2})}\right\}}^{r|k|}\\ & \leq & {\left(\frac{n\Lambda_{\left|k\right|}+\tau^{-1}}{n\lambda +\tau^{-1}}\right)}^{|k|/2}2^{r|k|-1}\left\{{\left(\frac{4V}{\left|k\right|}\right)}^{r|k|}+{\left(\frac{4{\left[(2r-1)!!\right]}^{\frac{1}{r}}}{n(\lambda +\tau^{-1})}\right)}^{r|k|}\right\}, \end{array}$$

where $V=\|{\beta_{k}}^{*}{\|}_{2}^{2}$, and

$$\begin{array}{c} \int_{B^{c}}\exp\left\{-\frac{{\beta_{k}}^{⊤}U_{k}\beta_{k}}{2\tau }\right\}\prod_{i=1}^{\left|k\right|}\beta_{k_{i}}^{2r}d\beta_{k}\leq {\left(C\tau \right)}^{|k|/2}, \end{array}$$

for some constant C>0. Further note that

$$\begin{array}{ccc} \det{\left\{H_{n}\left(\beta_{0,k}\right)\left(1-\epsilon \right)+\tau^{-1}I\right\}}^{1/2} & \leq & {\left(n\Lambda_{\left|k\right|}+\tau^{-1}\right)}^{|k|/2}\\ & \leq & \exp\left\{C|k|\log n\right\}\\ & \ll & \exp\left\{\frac{1-\epsilon }{8}c^{2}\lambda \left|k\right|\Lambda_{\left|k\right|}\log p\right\} \end{array}$$

for some constant C>0 and some large constant c>0, by Conditions (A1), (A2) and (A4). Therefore, it follows from (A3) that

(A4) $$\begin{array}{ccc} m_{k}\left(y\right) & \leq & C^{-|k|/2}{\left(\tau \right)}^{-r|k|-|k|/2}\exp\left\{L_{n}\left({\hat{\beta }}_{k}\right)\right\}\det{\left\{H_{n}\left(\beta_{0,k}\right)\left(1-\epsilon \right)+\tau^{-1}U_{k}\right\}}^{-1/2}\\ & & \times \left[{\Lambda_{\left|k\right|}}^{|k|/2}\left\{{\left(\frac{V}{\left|k\right|}\right)}^{r|k|}+n^{-r|k|}\right\}+o\left(1\right)\right]\\ & \leq & C^{-|k|/2}{\left(\tau \right)}^{-r|k|-|k|/2}\exp\left\{L_{n}\left({\hat{\beta }}_{k}\right)\right\}\det{\left\{H_{n}\left(\beta_{0,k}\right)\left(1-\epsilon \right)+\tau^{-1}U_{k}\right\}}^{-1/2}\\ & & \times {\Lambda_{\left|k\right|}}^{|k|/2}\left\{{\left(\frac{V}{\left|k\right|}\right)}^{r|k|}+n^{-r|k|}\right\}, \end{array}$$

for some constant C>0. Next, note that it follows from Lemma A.3 in the supplementary material for [5] that

$$\begin{array}{ccc} V=\|{\beta_{k}}^{*}{\|}_{2}^{2}\leq {\left\|{\hat{\beta }}_{k}\right\|}_{2}^{2} & \leq & {\left(\|{\hat{\beta }}_{k}-\beta_{0,k}{\|}_{2}+{\left\|\beta_{0,k}\right\|}_{2}\right)}^{2}\\ & \leq & {\left(\sqrt{\frac{\left|k\right|\Lambda_{\left|k\right|}\log p}{n}}+\sqrt{\log p}\right)}^{2}\\ & \leq & 2\left(\frac{\left|k\right|\Lambda_{\left|k\right|}\log p}{n}+\log p\right). \end{array}$$

Therefore,

$$\begin{array}{ccc} {\left(\frac{V}{\left|k\right|}\right)}^{r|k|} & \leq & {\left(\frac{2\log p}{\left|k\right|}\right)}^{r|k|}\exp\left({r|k|}^{2}\frac{\Lambda_{\left|k\right|}}{n}\right)\\ & ≲ & {\left(\frac{2\log p}{\left|k\right|}\right)}^{r|k|} \end{array}$$

by Conditions (A1) and (A2). Combining with (A4), we obtain the following upper bound for $m_{k}\left(y\right)$,

(A5) $$\begin{array}{ccc} m_{k}\left(y\right) & \leq & {\left(C_{1}\tau \right)}^{-r|k|-|k|/2}\exp\left\{L_{n}\left({\hat{\beta }}_{k}\right)\right\}\det{\left\{H_{n}\left(\beta_{0,k}\right)\left(1-\epsilon \right)+\tau^{-1}U_{k}\right\}}^{-1/2}\\ & & \times {\Lambda_{\left|k\right|}}^{|k|/2}{\left(\frac{\log p}{\left|k\right|}\right)}^{r|k|}, \end{array}$$

for any $k\in M_{1}$ and some constant $C_{1}>0$. Similarly, by Lemma 4 in the supplementary material for [8] and the similar arguments leading up to (A5), with probability tending to 1, we have

$$\begin{array}{ccc} m_{t}\left(y\right) & ≳ & {\left(C_{1}\tau \right)}^{-r|t|-|t|/2}\exp\left\{L_{n}\left({\hat{\beta }}_{t}\right)\right\}\det{\left\{H_{n}\left(\beta_{0,t}\right)\left(1+\epsilon \right)+\tau^{-1}U_{t}\right\}}^{-1/2}\\ & & \times \;\;\exp\left\{-\frac{1}{2}{\hat{\beta }}_{t}^{⊤}\left(A_{t}-A_{t}{\left(A_{t}+\tau^{-2}I_{t}\right)}^{-1}A_{t}\right){\hat{\beta }}_{t}\right\}{\left(\log p\right)}^{-r|t|}\\ & ≳ & {\left(C_{1}\tau \right)}^{-r|t|-|t|/2}\exp\left\{L_{n}\left({\hat{\beta }}_{t}\right)\right\}\det{\left\{H_{n}\left(\beta_{0,t}\right)\left(1+\epsilon \right)+\tau^{-1}U_{t}\right\}}^{-1/2}{\left(\log p\right)}^{-r|t|} \end{array}$$

by Lemma A1, where $A_{t}=\left(1+\epsilon \right)H_{n}\left(\beta_{0,t}\right)$. Therefore, with probability tending to 1,

(A6) $$\begin{array}{ccc} \frac{m_{k}\left(y\right)}{m_{t}\left(y\right)} & ≲ & {\left\{C_{1}n^{1/2}\tau^{r+1/2}\right\}}^{-(|k|-|t|)}\frac{\det{\left\{n^{-1}H_{n}\left(\beta_{0,t}\right)\left(1+\epsilon \right)+{\left(n\tau \right)}^{-1}U_{t}\right\}}^{1/2}}{\det{\left\{n^{-1}H_{n}\left(\beta_{0,k}\right)\left(1-\epsilon \right)+{\left(n\tau \right)}^{-1}U_{k}\right\}}^{1/2}}\\ & & \times \;\;\exp\left\{L_{n}\left({\hat{\beta }}_{k}\right)-L_{n}\left({\hat{\beta }}_{t}\right)\right\}{\Lambda_{\left|k\right|}}^{\frac{\left|k\right|}{2}}{\left(\frac{\log p}{\left|k\right|}\right)}^{r|k|}{\left(\log p\right)}^{r|t|}\\ & ≲ & {\left\{C_{1}p^{2+\delta }\right\}}^{-(|k|-|t|)}{\left(\frac{2}{\lambda }\right)}^{\left|k|-|t\right|}\exp\left\{L_{n}\left({\hat{\beta }}_{k}\right)-L_{n}\left({\hat{\beta }}_{t}\right)\right\}{\left(\log p\right)}^{r(2|k|+|t|)}\\ & ≲ & {\left(C_{1}p\right)}^{-(2+\delta )(|k|-|t|)}{\left(\frac{2}{\lambda }\right)}^{\left|k|-|t\right|}p^{\left(1+\delta^{*}\right)\left(1+2w\right)\left(|k\right|-\left|t|\right)}{\left(\log p\right)}^{r(2|k|+|t|)} \end{array}$$

for any $k\in M_{1}$, where the second inequality holds by Lemma 2 in [38], Conditions (A2) and (A4), and the third inequality follows from Lemma 3 in [38], which implies

(A7) $$\begin{array}{ccc} L_{n}\left({\hat{\beta }}_{k}\right)-L_{n}\left({\hat{\beta }}_{t}\right) & \leq & b_{n}\left(|k\right|-\left|t|\right) \end{array}$$

for any $k\in M_{1}$ with probability tending to 1, where $b_{n}=\left(1+\delta^{*}\right)\left(1+2w\right)\log p$ with some small constant w>0 satisfying $1+\delta >\left(1+\delta^{*}\right)\left(1+2w\right)$. Hence, with probability tending to 1, it follows from (A6) that

(A8) ∑k∈M1PR(k,t)=∑k⊋tπ(k)mk(y)π(t)mt(y)≤∑k⊋tmk(y)mt(y)≤∑|k|−|t|=1mn−|t|p−|t||k|−|t|p−(1+c)(|k|−|t|).

for some constant c>0. Using p−|t||k|−|t|≤p|k|−|t| and (A8), we get

$$\begin{array}{c} \sum_{k\in M_{1}}PR\left(k,t\right)\;\;=\;\;o\left(1\right). \end{array}$$

Thus, we have proved the desired result (A2). □

**Proof of Theorem** **2.** Let $M_{2}=\{k:k⊉t,|k|\leq m_{n}\}$. For any $k\in M_{2}$, let $k^{*}=k\cup t$, so that $k^{*}\in M_{1}$. Let $\beta_{k^{*}}$ be the $|k^{*}|$-dimensional vector including $\beta_{k}$ for k and zeros for t∖k. Then by Taylor’s expansion and Lemmas A.1 and A.3 in [5], with probability tending to 1,

$$\begin{array}{ccc} L_{n}\left(\beta_{k^{*}}\right) & = & L_{n}\left({\hat{\beta }}_{k^{*}}\right)-\frac{1}{2}{\left(\beta_{k^{*}}-{\hat{\beta }}_{k^{*}}\right)}^{⊤}H_{n}\left({\tilde{\beta }}_{k^{*}}\right)\left(\beta_{k^{*}}-{\hat{\beta }}_{k^{*}}\right)\\ & \leq & L_{n}\left({\hat{\beta }}_{k^{*}}\right)-\frac{1-\epsilon }{2}{\left(\beta_{k^{*}}-{\hat{\beta }}_{k^{*}}\right)}^{⊤}H_{n}\left(\beta_{0,k^{*}}\right)\left(\beta_{k^{*}}-{\hat{\beta }}_{k^{*}}\right)\\ & \leq & L_{n}\left({\hat{\beta }}_{k^{*}}\right)-\frac{n(1-\epsilon )\lambda }{2}{\left\|\beta_{k^{*}}-{\hat{\beta }}_{k^{*}}\right\|}_{2}^{2} \end{array}$$

for any $\beta_{k^{*}}$ such that $\|\beta_{k^{*}}-\beta_{0,k^{*}}{\|}_{2}\leq c\sqrt{|k^{*}|\Lambda_{|k^{*}|}\log p/n}=cw_{n}$ for some large constant c>0. Please note that Let $B_{k}=n\left(1-\epsilon \right)\lambda I_{k}$ and ${\beta_{k}}^{*}={\left(B_{k}+U_{k}/\tau \right)}^{-1}B_{k}{\hat{\beta }}_{k}$,

$$\begin{array}{cc} & \int \exp\left\{-\frac{n(1-\epsilon )\lambda }{2}{\left\|\beta_{k^{*}}-{\hat{\beta }}_{k^{*}}\right\|}_{2}^{2}\right\}\exp\left(-\frac{{\beta_{k}}^{⊤}U_{k}\beta_{k}}{2\tau }\right)\prod_{i=1}^{\left|k\right|}\beta_{k_{i}}^{2r}d\beta_{k}\\ = & \int \exp\left\{-\frac{n(1-\epsilon )\lambda }{2}{\left\|\beta_{k}-{\hat{\beta }}_{k}\right\|}_{2}^{2}-\frac{1}{2\tau }{\beta_{k}}^{⊤}U_{k}\beta_{k}\right\}\prod_{i=1}^{\left|k\right|}\beta_{k_{i}}^{2r}d\beta_{k}\;\exp\left\{-\frac{n(1-\epsilon )\lambda }{2}{\left\|{\hat{\beta }}_{t\setminus k}\right\|}_{2}^{2}\right\}\\ = & {\left(2\pi \right)}^{\frac{\left|k\right|}{2}}{\left|B_{k}+U_{k}/\tau \right|}^{-1/2}\exp\left\{-\frac{1}{2}{\hat{\beta }}_{k}^{⊤}\left(B_{k}-B_{k}{\left(B_{k}+U_{k}/\tau \right)}^{-1}B_{k}\right){\hat{\beta }}_{k}\right\}E_{k}\left(\prod_{i=1}^{\left|k\right|}\beta_{k_{i}}^{2r}\right)\\ & \times \;\;\exp\left\{-\frac{n(1-\epsilon )\lambda }{2}{\left\|{\hat{\beta }}_{t\setminus k}\right\|}_{2}^{2}\right\}. \end{array}$$

where $E_{k}\left(.\right)$ denotes the expectation with respect to a multivariate normal distribution with mean ${\beta_{k}}^{*}$ and covariance matrix ${\left(B_{k}+U_{k}/\tau \right)}^{-1}$. It follows from Lemma 6 in the supplementary material for [8] that

$$\begin{array}{ccc} E_{k}\left(\prod_{i=1}^{\left|k\right|}\beta_{k_{i}}^{2r}\right) & \leq & {\left(\frac{n\lambda +\tau^{-1}}{n\lambda +\tau^{-1}}\right)}^{|k|/2}{\left\{\frac{4V}{\left|k\right|}+\frac{4{\left[(2r-1)!!\right]}^{\frac{1}{r}}}{n(\lambda +\tau^{-1})}\right\}}^{r|k|}\\ & \leq & {\left(\frac{n\lambda +\tau^{-1}}{n\lambda +\tau^{-1}}\right)}^{|k|/2}2^{r|k|-1}\left\{{\left(\frac{4V}{\left|k\right|}\right)}^{r|k|}+{\left(\frac{4{\left[(2r-1)!!\right]}^{\frac{1}{r}}}{n(\lambda +\tau^{-1})}\right)}^{r|k|}\right\}, \end{array}$$

where $V=\|{\beta_{k}}^{*}{\|}_{2}^{2}$. Define the set $B_{*}:=\left\{\beta_{k}:{\left\|\beta_{k^{*}}-{\hat{\beta }}_{k^{*}}\right\|}_{2}\leq cw_{n}/2\right\},$ for some large constant c>0, then by similar arguments used for super sets, with probability tending to 1,

$$\begin{array}{ccc} \pi (k|y) & = & d_{k}{\left(2\pi \right)}^{-|k|/2}{\left(\tau \right)}^{-r|k|-|k|/2}{\left|U_{k}\right|}^{\frac{1}{2}}\int_{B_{*}\cup B_{*}^{c}}\exp\left\{L_{n}\left(\beta_{G_{k^{*}}}\right)\right\}\exp\left(-\frac{{\beta_{k}}^{⊤}U_{k}\beta_{k}}{2\tau }\right)\prod_{i=1}^{\left|k\right|}\beta_{k_{i}}^{2r}d\beta_{k}\\ & ≲ & {\left(C_{1}\tau \right)}^{-r|k|-\left|k\right|/2}\exp\left\{L_{n}\left({\hat{\beta }}_{k^{*}}\right)\right\}\det{\left(B_{k}+U_{k}/\tau \right)}^{-1/2}\\ & & \times \left[\exp\left\{-\frac{n(1-\epsilon )\lambda }{2}{\left\|{\hat{\beta }}_{t\setminus k}\right\|}_{2}^{2}\right\}{\left(\frac{\log p}{\left|k\right|}\right)}^{r|k|}+\exp\left\{-cC|k^{*}|\Lambda_{|k^{*}|}\log p\right\}\right] \end{array}$$

for any $k\in M_{2}$ and for some constant C>0. Since the lower bound for π(t|y) can be derived as before, it leads to

(A9) $$\begin{array}{ccc} PR(k,t) & ≲ & {\left\{C_{1}n^{1/2}\tau^{r+1/2}\right\}}^{-(|k|-|t|)}\frac{\det{\left\{\left(1+\epsilon \right)n^{-1}H_{n}\left(\beta_{0,t}\right)+{\left(n\tau \right)}^{-1}U_{t}\right\}}^{1/2}}{\det{\left\{\left(1-\epsilon \right)\lambda I_{k}+{\left(n\tau \right)}^{-1}U_{k}\right\}}^{1/2}}\\ & & \times \;\;\exp\left\{L_{n}\left({\hat{\beta }}_{k^{*}}\right)-L_{n}\left({\hat{\beta }}_{t}\right)\right\}\;\exp\left\{-\frac{n(1-\epsilon )\lambda }{2}{\left\|{\hat{\beta }}_{t\setminus k}\right\|}_{2}^{2}\right\}{\left(\frac{\log p}{\left|k\right|}\right)}^{r|k|}{\left(\log p\right)}^{r|t|} \end{array}$$

(A10) $$\begin{array}{cc} + & {\left\{C_{1}n^{1/2}\tau^{r+1/2}\right\}}^{-(|k|-|t|)}\det{\left\{\left(1+\epsilon \right)n^{-1}H_{n}\left(\beta_{0,t}\right)+{\left(n\tau \right)}^{-1}U_{t}\right\}}^{1/2}\\ & \times \;\;\exp\left\{L_{n}\left({\hat{\beta }}_{k^{*}}\right)-L_{n}\left({\hat{\beta }}_{t}\right)\right\}\exp\left\{-c\;C|k^{*}|\Lambda_{|k^{*}|}\log p\right\}{\left(\log p\right)}^{r|t|} \end{array}$$

for any $k\in M_{2}$ with probability tending to 1. We first focus on (A9). Please note that

$$\begin{array}{cc} & \frac{\det{\left\{\left(1+\epsilon \right)n^{-1}H_{n}\left(\beta_{0,t}\right)+{\left(n\tau \right)}^{-1}U_{t}\right\}}^{1/2}}{\det{\left\{\left(1-\epsilon \right)\lambda I_{k}+{\left(n\tau \right)}^{-1}U_{k}\right\}}^{1/2}}\\ \leq & \frac{{\left\{\left(1+\epsilon \right)\Lambda_{\left|t\right|}+{\left(n\tau \right)}^{-1}\right\}}^{|t|/2}}{{\left\{\left(1-\epsilon \right)\lambda +{\left(n\tau \right)}^{-1}\right\}}^{|k|/2}}\\ = & {\left\{\frac{\left(1+\epsilon \right)\Lambda_{\left|t\right|}+{\left(n\tau \right)}^{-1}}{\left(1-\epsilon \right)\lambda +{\left(n\tau \right)}^{-1}}\right\}}^{|t|/2}{\left\{\frac{1}{\left(1-\epsilon \right)\lambda +{\left(n\tau \right)}^{-1}}\right\}}^{(|k|-|t|)/2}\\ ≲ & \exp\left\{C|t|\log\Lambda_{\left|t\right|}\right\}\;{\left\{\frac{1}{\left(1-\epsilon \right)\lambda +{\left(n\tau \right)}^{-1}}\right\}}^{(|k|-|t|)/2} \end{array}$$

for some constant C>0. Furthermore, by the same arguments used in (A7), we have

$$\begin{array}{ccc} L_{n}\left({\hat{\beta }}_{k^{*}}\right)-L_{n}\left({\hat{\beta }}_{t}\right) & ≲ & C_{*}\left(\right|k^{*}|-|t|)\log p\\ & = & C_{*}|t\setminus k|\log p+C_{*}\left(|k\right|-|t|)\log p \end{array}$$

for some constant $C_{*}>0$ and for any $k\in M_{2}$ with probability tending to 1. Here we choose $C_{*}=\left(1+\delta^{*}\right)\left(1+2w\right)$ if |k|>|t| or $C_{*}=3+\delta$ if |k|≤|t| so that

$$\begin{array}{cc} & {\left\{C_{1}n^{1/2}\tau^{r+1/2}\right\}}^{-(|k|-|t|)}{\left\{\frac{1}{\left(1-\epsilon \right)\lambda +{\left(n\tau \right)}^{-1}}\right\}}^{(|k|-|t|)/2}p^{C_{*}\left(|k\right|-\left|t|\right)}\\ & \times \;\;\exp\left\{r|k|\log\left(\frac{\log p}{\left|k\right|}\right)+r\left|t\right|\log\left(\log p\right)\right\}\\ ≲ & p^{\left(C_{*}-2-\delta \right)\left(|k\right|-\left|t|\right)}\;\;=\;\;o\left(1\right), \end{array}$$

where the inequality holds by Condition (A4). To be more specific, we divide $M_{2}$ into two disjoint sets $M_{2}^{\prime }=\{k:k\in M_{2},|t|<|k|\leq m_{n}\}$ and $M_{2}^{*}=\{k:k\in M_{2},|k|\leq \left|t|\right\}$, and will show that $\sum_{k\in M_{2}^{\prime }}PR\left(k,t\right)+\sum_{k\in M_{2}^{*}}PR\left(k,t\right)⟶0$ as n→∞ with probability tending to 1. Thus, we can choose different $C_{*}$ for $M_{2}^{\prime }$ and $M_{2}^{*}$ as long as $C_{*}\geq \left(1+\delta^{*}\right)\left(1+2w\right)$. On the other hand, with probability tending to 1, by Condition (A3),

$$\begin{array}{ccc} \exp\left\{-\frac{n(1-\epsilon )\lambda }{2}{\left\|{\hat{\beta }}_{t\setminus k}\right\|}_{2}^{2}\right\} & \leq & \exp\left[-\frac{n(1-\epsilon )\lambda }{2}\left\{\|\beta_{0,t\setminus k}{\|}_{2}^{2}-{\left\|{\hat{\beta }}_{t\setminus k}-\beta_{0,t\setminus k}\right\|}_{2}^{2}\right\}\right]\\ & \leq & \exp\left[-\frac{n(1-\epsilon )\lambda }{2}\left\{{\left|t\setminus k\right|}^{2}\min_{j\in t}\beta_{0,j}^{2}-c^{\prime }w_{n}^{\prime 2}\right\}\right]\\ & \leq & \exp\left\{-\frac{(1-\epsilon )\lambda }{2}\left(c_{0}{\left|t\setminus k\right|}^{2}\left|t\right|-c^{\prime }\left|t\setminus k\right|\right)\Lambda_{\left|t\right|}\log p\right\}\\ & \leq & \exp\left\{-\frac{(1-\epsilon )\lambda }{2}\left(c_{0}-c^{\prime }\right){\left|t\setminus k\right|}^{2}\left|t\right|\Lambda_{\left|t\right|}\log p\right\} \end{array}$$

for any $k\in M_{2}$ and some large constants $c_{0}>c^{\prime }>0$, where $w_{n}^{\prime 2}=\left|t\setminus k\right|\Lambda_{\left|t\setminus k\right|}\log p/n$. Here, $c^{\prime }=5\lambda^{-2}{\left(1-\epsilon \right)}^{-2}$ by the proof of Lemma A.3 in [5]. Hence, (A9) for any $k\in M_{2}$ is bounded above by

$$\begin{array}{cc} & \exp\left\{|t|\log\Lambda_{\left|t\right|}+C_{*}|t\setminus k|\log p-\frac{(1-\epsilon )\lambda }{2}\left(c_{0}-c^{\prime }\right){\left|t\setminus k\right|}^{2}\left|t\right|\Lambda_{\left|t\right|}\log p\right\}\\ ≲ & \exp\left\{-\left(\frac{(1-\epsilon )\lambda }{2}\left(c_{0}-c^{\prime }\right)-C_{*}-o\left(1\right)\right){\left|t\setminus k\right|}^{2}\left|t\right|\Lambda_{\left|t\right|}\log p\right\}\\ \leq & \exp\left\{-\left(\frac{(1-\epsilon )\lambda }{2}\left(c_{0}-c^{\prime }\right)-C_{*}-o\left(1\right)\right){\left|t\setminus k\right|}^{2}\left|t\right|\Lambda_{\left|t\right|}\log p\right\}\\ \leq & \exp\left\{-\left(\frac{(1-\epsilon )\lambda }{2}\left(c_{0}-c^{\prime }\right)-C_{*}-o\left(1\right)\right)\left|t\right|\Lambda_{\left|t\right|}\log p\right\} \end{array}$$

with probability tending to 1, where the last term is of order o(1) because we assume $c_{0}=\frac{1}{(1-\epsilon_{0})\lambda }\left\{2\left(3+\delta \right)+\frac{5}{(1-\epsilon_{0})\lambda }\right\}>\frac{2}{(1-\epsilon )\lambda }\left(C_{*}+o\left(1\right)\right)+c^{\prime }$ for some small $\epsilon_{0}>0$. It is easy to see that the maximum (A10) over $k\in M_{2}$ is also of order o(1) with probability tending to 1 by the similar arguments. Since we have (A2) in the proof of Theorem 1, it completes the proof. □

**Proof of Theorem** **3.** Let $M_{2}=\{k:k⊉t,|k|\leq m_{n}\}$. Since we have Theorem 1, it suffices to show that

(A11) $$\begin{array}{ccc} \sum_{k:k\in M_{2}}PR\left(k,t\right) & \overset{P}{⟶} & 0\;\;\mathrm{as}\;n\to \infty . \end{array}$$

By the proof of Theorem 2, the summation of (A9) over $k\in M_{2}$ is bounded above by

∑k∈M2p(C*−2−δ)(|k|−|t|)exp−(1−ϵ)λ2(c0−c′)−C*−o(1)|t∖k|2|t|Λ|t|logp≤∑|k|=0r∑v=0(|t|−1)∧|k||t|vr−|t||k|−vp−(|k|−|t|)exp−(1−ϵ)λ2(c0−c′)−C*−o(1)(|t|−v)2|t|Λ|t|logp≤∑|k|=0r∑v=0(|t|−1)∧|k||t|r|t|−vexp−(1−ϵ)λ2(c0−c′)−C*−o(1)(|t|−v)2|t|Λ|t|logp≤exp−(1−ϵ)λ2(c0−c′)−C*−o(1)|t|Λ|t|logp+2(|t|+2)logp≤exp−(1−ϵ)λ2(c0−c′)−C*−6−o(1)|t|Λ|t|logp

with probability tending to 1, where $C_{*}\leq 3+\delta$ is defined in the proof of Theorem 2. Please note that the last term is of order o(1) because we assume $c_{0}=\frac{1}{(1-\epsilon_{0})\lambda }\left\{2\left(9+2\delta \right)+\frac{5}{(1-\epsilon_{0})\lambda }\right\}>\frac{2}{(1-\epsilon )\lambda }\left(C_{*}+6+o\left(1\right)\right)+c^{\prime }$ for some small $\epsilon_{0}>0$. It is easy to see that the summation of (A10) over $k\in M_{2}$ is also of order o(1) with probability tending to 1 by the similar arguments. □

**Lemma** **A1.** *Under Condition* (A2)*, we have*

$$\begin{array}{ccc} \exp\left\{\frac{1}{2}{\hat{\beta }}_{k}^{⊤}\left(A_{k}-A_{k}{\left(A_{k}+\tau^{-1}U_{k}\right)}^{-1}A_{k}\right){\hat{\beta }}_{k}\right\} & ≲ & 1 \end{array}$$

*for any $k\in M_{1}$ with probability tending to 1.*

**Proof.** Please note that by Condition (A2),

$$\begin{array}{ccc} {\left(A_{k}+\tau^{-1}U_{k}\right)}^{-1} & \geq & {\left(A_{t}+{\left(n\tau \lambda \right)}^{-1}A_{k}\right)}^{-1}\;\;=\;\;\frac{n\tau \lambda }{n\tau \lambda +1}A_{k}^{-1}, \end{array}$$

which implies that

$$\begin{array}{ccc} \frac{1}{2}{\hat{\beta }}_{k}^{⊤}\left(A_{k}-A_{k}{\left(A_{k}+\tau^{-1}U_{k}\right)}^{-1}A_{k}\right){\hat{\beta }}_{k} & \leq & \frac{1}{2(n\tau \lambda +1)}{\hat{\beta }}_{k}^{⊤}A_{k}{\hat{\beta }}_{k}. \end{array}$$

Thus, we complete the proof if we show that

$$\begin{array}{ccc} \frac{1}{n\tau \lambda }{\hat{\beta }}_{k}^{⊤}H_{n}\left(\beta_{0,k}\right){\hat{\beta }}_{k} & \leq & C \end{array}$$

for some constant C>0 and any $k\in M_{1}$ with probability tending to 1. By Lemma A.3 in [5] and Condition (A2),

$$\begin{array}{ccc} \frac{1}{n\tau }{\hat{\beta }}_{k}^{⊤}H_{n}\left(\beta_{0,k}\right){\hat{\beta }}_{k} & \leq & \frac{1}{\tau }\lambda_{\max}\left\{n^{-1}H_{n}\left(\beta_{0,k}\right)\right\}{\left\|{\hat{\beta }}_{k}\right\|}_{2}^{2}\\ & \leq & \frac{1}{\tau }{\left(\log p\right)}^{d}\left(\|\beta_{0,k}{\|}_{2}^{2}+o\left(1\right)\right)\;\;=\;\;O\left(1\right) \end{array}$$

for any $k\in M_{1}$ with probability tending to 1. □
